# Actin as Deathly Switch? How Auxin Can Suppress Cell-Death Related Defence

**DOI:** 10.1371/journal.pone.0125498

**Published:** 2015-05-01

**Authors:** Xiaoli Chang, Michael Riemann, Qiong Liu, Peter Nick

**Affiliations:** 1 Department of Plant Pathology, Agricultural College, Sichuan Agricultural University, Chengdu 611130, P. R. China; 2 Molecular Cell Biology, Botanical Institute, Karlsruhe Institute of Technology, Kaiserstr. 2, D-76128 Karlsruhe, Germany; Ohio State University, UNITED STATES

## Abstract

Plant innate immunity is composed of two layers – a basal immunity, and a specific effector-triggered immunity, which is often accompanied by hypersensitive cell death. Initiation of cell death depends on a complex network of signalling pathways. The phytohormone auxin as central regulator of plant growth and development represents an important component for the modulation of plant defence. In our previous work, we showed that cell death is heralded by detachment of actin from the membrane. Both, actin response and cell death, are triggered by the bacterial elicitor harpin in grapevine cells. In this study we investigated, whether harpin-triggered actin bundling is necessary for harpin-triggered cell death. Since actin organisation is dependent upon auxin, we used different auxins to suppress actin bundling. Extracellular alkalinisation and transcription of defence genes as the basal immunity were examined as well as cell death. Furthermore, organisation of actin was observed in response to pharmacological manipulation of reactive oxygen species and phospholipase D. We find that induction of defence genes is independent of auxin. However, auxin can suppress harpin-induced cell death and also counteract actin bundling. We integrate our findings into a model, where harpin interferes with an auxin dependent pathway that sustains dynamic cortical actin through the activity of phospholipase D. The antagonism between growth and defence is explained by mutual competition for signal molecules such as superoxide and phosphatidic acid. Perturbations of the auxin-actin pathway might be used to detect disturbed integrity of the plasma membrane and channel defence signalling towards programmed cell death.

## Introduction

Animals use specific organs to fulfil specific functions. Plants lack such specialised organs, but instead employ cells that are highly flexible in terms of function. Whereas mobile defence cells constitute the core of animal immunity, plant defence is rather based upon the innate immunity of individual cells. This innate immunity derives from two layers [1]. The evolutionarily ancient PAMP-triggered immunity (PTI) is triggered upon recognition of conserved pathogen structures, so called pathogen-associated molecular patterns (PAMPs) through specific receptors on the plasma membrane. Biotrophic pathogens that are specialised to a specific host, have often evolved effectors that enter the cytoplasm of the host cell to quell the defence signalling triggered by the PAMP-receptors as a prerequisite of a biotrophic lifestyle [2]. As strategy against such advanced pathogens, plants have evolved additional pathogen-specific receptors (encoded by so-called R genes) that specifically recognise the effectors in the cytoplasm and reinstall defence signalling leading to a second layer of defence, so called effector-triggered immunity (ETI) [3]. Often, ETI culminates in a hypersensitive response, a plant-specific version of programmed cell death. Although the difference between PTI and ETI is less discrete than previously thought, this conceptual dichotomy has been very useful to classify the huge variety of plant defence responses.

To elicit the cellular events related to ETI-like programmed cell death, harpin proteins have been useful. These bacterial proteins were first discovered in *Erwinia amylovora*, a phytopathogenic bacterium causing the fire-blight disease of apple and pears [4], and are components of a bacterial type-III secretion system that can elicit host responses normally observed during the defence against biotrophic pathogens, such as oxidative burst, accumulation of defence-related transcripts, and cell death [5]. In our previous work, we have used a commercial preparation of harpin N to induce the full repertory of ETI-related responses in suspension cells of grapevine initiating with apoplastic alkalinisation, and oxidative burst, followed by activation of a MAPK cascade, cytoskeletal reorganisation, and the induction of defence genes, leading to the production of toxic phytoalexins, and terminating in cell death [6–8].

The cell death triggered by harpin elicitors is preceded by a rapid and specific reorganisation of the actin cytoskeleton: The cortical actin filaments subtending the cell membrane detach, and the entire actin skeleton contracts into dense cables towards the nucleus (*Vitis rupestris* in response to harpin N [6]; tobacco BY-2 in response to harpin Z [9]; *Arabidopsis* in response to flg22 [10,11]). A role of actin reorganisation for the induction of programmed cell death, a phenomenon progressively emerging for eukaryotic cells in general [12,13], has also been demonstrated for plant cells [14]. For instance, the bundling of actin cables in cells of the embryonic suspensor is not only a manifestation of ensuing cell death, but has been shown to be necessary and sufficient to initiate apoptosis in this system [15]

However, actin bundling does not necessarily result in cell death, but is also a typical feature of cells that have terminated (or failed to initiate) elongation growth. In response to auxin, actin bundles can be rapidly dissociated into fine strands, and growth resumes [16]. The fine actin strands formed in response to auxin will, in turn, stimulate the efflux of auxin, probably by modulating the cycling of auxin-efflux transporters between cytoplasm and the plasma membrane. The resulting alterations in the efflux of auxin will, in turn, alter the organisation of actin filaments, probably through modulation of actin-depolymerisation factor 2 [17], thus constituting a self-referring regulatory circuit.

This actin-auxin circuit might be relevant for the antagonistic relationship between defence and growth. The evolutionary background for this antagonism is to allocate resources otherwise used for growth or defence [18]. In fact, when defence-related traits are genetically impaired, this results in higher growth rates [19]. The defence-related bundling of actin filaments might therefore mediate an immediate arrest of cell growth, thus releasing all cellular resources towards defence. On the other hand, auxin might, through dissociation of actin bundles into finer filaments, modulate defence or even relocate cellular resources towards growth.

Prompted by these considerations we investigated, whether auxin can regulate defence responses elicited by harpin N in grapevine cells. We observe that apoplastic alkalinisation, the induction of defence genes, the reorganisation of actin filaments, and cell death can be modulated by natural and artificial auxins in a manner that is specific with respect to dose-dependency and transport properties of the respective auxin. We also observe that the modulating effect of auxin upon cell death can be phenocopied by a mild treatment with Latrunculin B (a compound sequestering G-actin). In addition, microscopical analysis of cortical actin in response to pharmacological manipulation shows that this dynamic population of actin is modulated by superoxide anions and phospholipase D. Using malone dialdehyde (MDA) as readout for superoxide (generated by the NADPH oxidase RboH), we show that auxin can quell harpin-induced oxidative burst. We discuss the data in the context of a model, where auxin, RboH, and actin are part of a switch between growth and death.

## Materials and Methods

### Cell culture and treatments

Cell suspension cultures of *Vitis rupestris* and *Vitis vinifera* cv. ‘Pinot Noir’ established from leaves were maintained in liquid MS medium containing 4.3 g l^-1^ Murashige and Skoog salts (Duchefa, Haarlem, Netherlands), 30 g l^-1^ sugar, 200 mg l^-1^ KH_2_PO_4_, 100 mg l^-1^ inositol, 1 mg l^-1^ thiamine, and 0.2 mg l^-1^ 2,4-dichlorophenoxy-acetic acid (2,4-D), pH 5.8. Cells were sub-cultured weekly by transferring 10 ml of stationary cells into 30 ml fresh medium in 100 ml Erlenmeyer flasks and incubated on an orbital shaker (KS250 basic, IKA Labortechnik, Germany) at 150 rpm and 25°C in the dark.

A commercially available harpin elicitor (Messenger, EDEN Bioscience Corporation, Washington, USA; 3% of active ingredient harpin protein) was dissolved in MS liquid medium to yield a stock solution of 300 mg ml^-1^ and administered in a concentration of 9 μg ml^-1^ [6]. Indole-3-acetic acid (IAA), α-Naphthalene acetic acid (NAA), and 2,4-dichlorophenoxyacetic acid (2,4-D) were dissolved in ethanol to yield stock solutions of 100 μM, respectively. To assess the role of actin filaments for cell viability, an inhibitor of actin polymerisation, Latrunculin B (Lat B, Sigma, Deisenhofen, Germany) was employed at 2 μM based on results of previous work in the same cell lines [7]. *n*-butanol, an inhibitor of phospholipase D, and its inactive analogue *sec*-butanol were diluted into culture medium as solvent and used for observation of actin dynamics. Diphenylene-iodonium chloride (DPI, Sigma-Aldrich, Deisenhofen, Germany), an inhibitor of NADPH oxidase, was prepared in dimethylsulfoxide (DMSO) to a stock solution of 10 mM. All treatments were accompanied by solvent controls, where the maximal concentration of solvent used in the test samples was administered and not exceeded 0.1%. All experiments were performed at day 4 after sub-cultivation, when the culture had completed proliferation and was undergoing cell expansion.

### Measurement of extracellular alkalinisation

Extracellular alkalinisation was measured by combining a pH meter (Schott handylab, pH 12) with a pH electrode (Mettler Toledo, LoT 403-M8-S7/120), and recorded by a paperless readout (VR06; MF Instruments GmbH, Albstadt-Truchtelfingen, Germany). Before addition of elicitors, cells were pre-adapted on an orbital shaker for at least 1 h. To test the effect of auxin on harpin-dependent extracellular alkalinisation, the naturally occuring auxin, IAA, and two synthetic auxins, NAA and 2,4-D, were applied. After adaptation, cells were inoculated with either ethanol as a solvent control, harpin as a positive control, auxins without harpin (either 10 μM or 50 μM of IAA, NAA, or 2,4-D), or a combination of harpin with auxins (IAA, NAA, or 2,4-D, respectively). The change of pH was recorded over time. The experiments were repeated at least five times.

### Analysis of gene expression

Total RNA was extracted from 1 ml of *V*. *rupestris* or cv. ‘Pinot Noir’ cells at day 4 after sub-cultivation using the RNeasy Plant Mini Kit (Qiagen, Hilden, Germany) or the Plant Total RNA Kit (Sigma, Deisenhofen, Germany), respectively, following the protocol of the producers. Gene expression was analysed 30 min after addition of harpin, preceded by incubation for 60 min with the different auxins. The extracted RNA was treated with a DNA-free DNase (Qiagen, Hilden, Germany) to remove potential contamination of genomic DNA. The mRNA was transcribed into cDNA using the M-MuLV cDNA Synthesis Kit (New England BioLabs; Frankfurt am Main, Germany) according to the instructions of the manufacturer. The RNase inhibitor (Invitrogen, Karlsruhe, Germany) was used to remove contamination by non-transcribed RNA. Transcription of the selected genes (*PAL*, *StSy*, *PR*5 and *PR*10) was tested by semi-quantitative reversible transcription PCR (RT-PCR) following 30 cycles of 30 s denaturation at 94°C, 30 s annealing at 60°C, and 1 min synthesis at 72°C using a conventional PCR cycler (peqLab Primus 96, Erlangen, Germany) using the primers referred to Kortekamp’s studies [20] (S1 Table). The bands of the products were quantified using the Image J software (http://rsbweb.nih.gov/ij/) and standardised relative to elongation factor 1α (EF 1 α) as internal standard [21]. The results were plotted as relative increase of transcript abundance as compared with the untreated control. The data represent mean and standard errors from at least three independent experimental series. Statistical significance was tested by ANOVA analysis.

### Determination of cell viability

To determine cell viability, cells were stained at different time points with Evans Blue [22]. Cells were transferred into a custom-made staining chamber [23] to remove the medium, and then incubated with 2.5% Evans Blue for 3–5 min. After washing three times with distilled water, cells were mounted on a slide and observed under a light microscope (Zeiss-Axioskop 2 FS, DIC illumination, 20 × objective). Due to the breakdown of the plasma membrane, Evans Blue is capable of penetrating into dead cells, resulting in a blue staining of the cell interior. Frequency of cell death was calculated as ratio of the number of dead cells over the total number of scored cells. For each time point, 1500 cells were scored in three dependent experiments.

### Visualisation of cytoskeletal actin filaments

Actin filaments were visualised by fluorescent phalloidin following the protocol published in Waller and Nick (1997) [24] with minor modifications. The principle of this protocol is to use a very mild fixation that will permeabilise the plasma membrane, but leaves the tonoplast mostly intact to avoid cytoplasmic coagulations. After fixation in 0.85% (w/v) fresh paraformaldehyde in microfilament buffer [100 mM potassium phosphate buffer, 100 mM KCl, 0.25% (v/v) Triton X100, pH 7.3] for 15 min, samples were washed three times for 5 min using a custom-made staining mesh [23], and then incubated in 130 nM fluorescein-isothiocyanate labelled phalloidin (Sigma, Deisenhofen, Germany) for 45 min. Subsequently, the specimens were washed additional three times just prior to mounting for microscopical observation. Confocal images were recorded with an AxioObserver Z1 (Zeiss, Jena, Germany) using a 63x LCI-Neofluar Imm Corr DIC objective (NA 1.3), the 488 nm emission line of an Ar-Kr laser, and a spinning-disc device (YOKOGAWA CSU-X1 5000). Apparent thickness of actin cables was quantified as described in Nick *et al*. (2009) [25]. In brief, projections of z-stacks over the cortical layer were probed using the density profiling tool of the Image J software (National Institute of Health) setting the width of the probing line to eight pixels to integrate over local differences of fluorescence intensity. A grid of five lines oriented perpendicular with the longer cell axis and spaced equally over its length were recorded and averaged for each cell. For each individual profile, a function f(d_i_) of the measured density d_i_ was calculated over pixel position with the equation as follow:

f(di)=di'(di) (1)

in the rising flank of an actin filament, this will produce a value of +1, in the dropping flank of an actin filament, this will produce a value of -1, in the interspace between two actin filaments, this will produce a value of 0. To discriminate random fluctuations of density from actin filaments, the resulting values were multiplied with their preceding value (f (d_i_) · f (d_i-1_)), which will reduce all random fluctuations to 0. In the next step, all values will be squared, such that all pixels covered by an actin filament will get a value of 1. The sum S over the entire profile will thus report the part of the profile that is covered by actin filaments. To determine the number of filaments, the first derivative of the entire row is formed—this will be -1 at the leading edge of a filament and +1 at its trailing edge. The number of filaments crossed by the profile can now be obtained by summing up the squares of these first derivatives and dividing them by a factor of 2 (because each filament has a leading and a trailing edge). The average thickness of actin filaments w can now be calculated as ratio of S and the number of filaments. For each cell, the values for 5 profiles are averaged.

### Analysis of lipid peroxidation

Lipid peroxidation as readout for oxidative burst was determined by measuring the reaction product Malondialdehyde (MDA) as described by Heath and Packer (1968) [26]. 2 ml of *Vitis* cells were treated with 9 μg ml^-1^ harpin for 30 min, or pretreated with 50 μM IAA for 1 h followed by harpin for 30 min using MS medium as control. Cells were sedimented and ground in 1 ml of ice-cold reagent [0.25% (w/v) 2- thiobarbituric acid (TBA) in 10% (w/v) trichloroacetic acid]. After incubation at 95°C for 20 min, the extracts were cooled at room temperature and then centrifuged at 12 000 rpm for 10 min. MDA-dependent conversion of TBA into a coloured adduct was determined in the supernatant by recording the absorbance at 532 nm as compared to non-specific compounds at 600 nm using a ultraviolet spectrophotometer (Uvicon).

## Results

### Auxin alters harpin-induced extracellular alkalinisation

It has been reported that auxin is linked to plant immunity [27,28], possibly connected with changes of cell wall structure accompanying alterations of apoplastic pH [29]. Hence, the effect of auxin on harpin-induced extracellular alkalinisation was investigated (S1 Fig). Most auxin responses showed a characteristic bell-shaped dose-response curve for the natural auxin IAA with an optimum at 10 μM, and a reduced effect at superoptimal concentrations (50 μM). Therefore, these two concentrations were selected.

**Fig 1:**
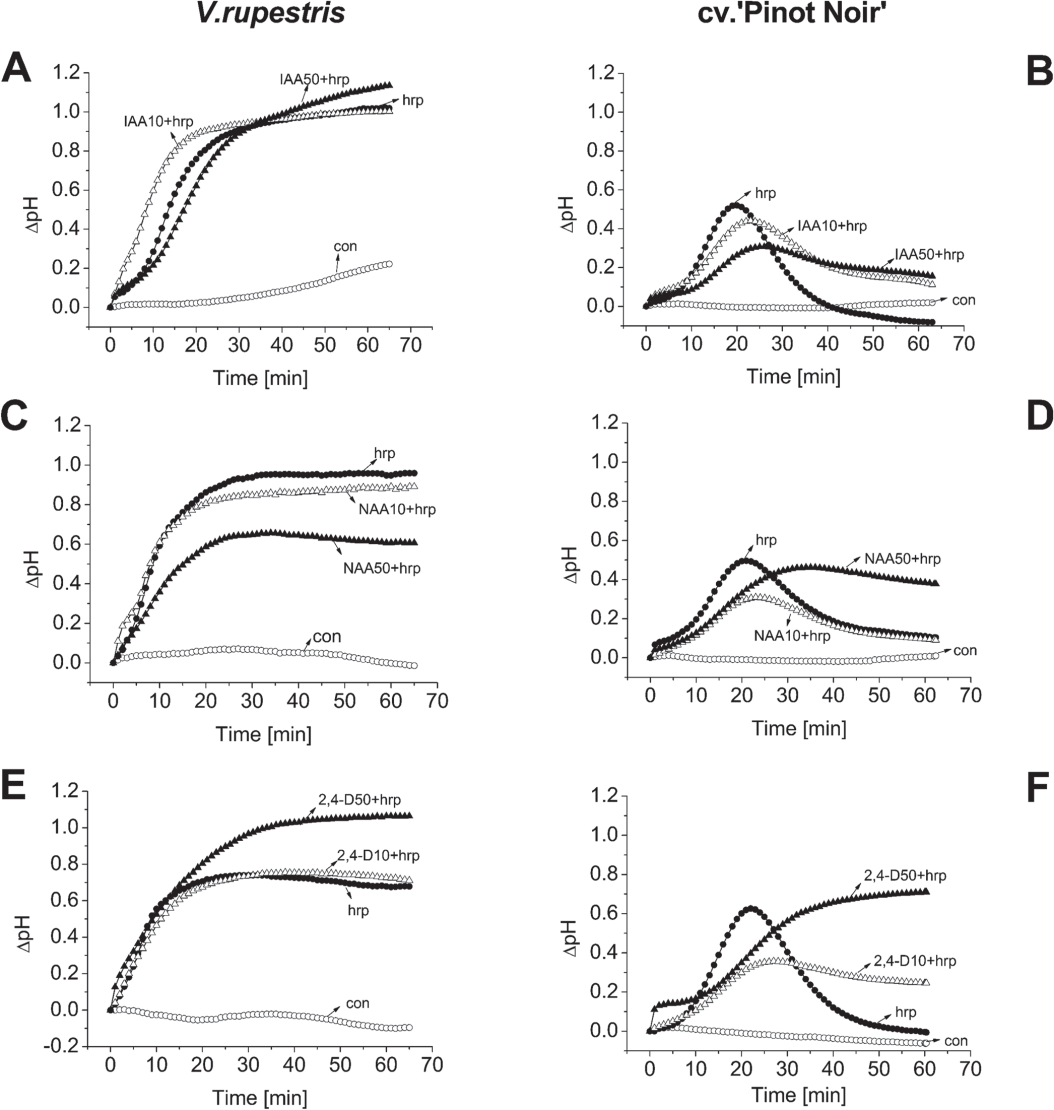
Modulation of harpin-triggered apoplastic alkalinisation by different auxins. Cells were treated with 9 μg ml^-1^ harpin (hrp, closed circles) as a positive control, harpin combined with 10 μM (open triangles) or 50 μM auxins (IAA, NAA, or 2, 4-D, closed triangles), or ethanol used as solvent control (con, open circles) in *V*. *rupestris* (**A**, **C**, and **E**) and cv. ‘Pinot Noir’ (**B**, **D**, and **F**). Representative experiments from five biological replicas are depicted. Harpin and auxin were added at time 0, if measured isolated, for the combinations, auxins were added 1 h prior to harpin.

As Fig 1 shown, in *V*. *rupestris*, 10 μM of the natural auxin IAA promoted alkalinisation slightly, but significantly, whereas the superoptimal concentration (50 μM) delayed the response (Fig 1A). In cv. ‘Pinot Noir’, alkalinisation initiated at the same time, but was increasing with a reduced slope, followed by a constitutively elevated pH (Fig 1B). Here, the auxin effect was more pronounced for the high concentration. For the stable artificial auxin NAA, the alkalinisation response in *V*. *rupestris* was inhibited already at 10 μM, and this inhibition was raised even further at 50 μM (Fig 1C). For cv. ‘Pinot Noir’, the reduction in slope of the response and the subsequent stable elevation of pH were stronger as compared to IAA (Fig 1D). The non-transportable artificial auxin 2,4-D did not accelerate the response in *V*. *rupestris*, but increased its amplitude (Fig 1E), whereas in cv. ‘Pinot Noir’, the stable elevation of pH was even further amplified over that observed for NAA (Fig 1F). Thus, in cv. ‘Pinot Noir’, auxins do not alter the onset of the alkalinisation response, but slow its development and cause a stable increase of pH depending on their stability and transportability. In contrast, in *V*. *rupestris*, the natural auxin IAA accelerated the response, whereas NAA and 2, 4-D just changed its amplitude (NAA negatively, 2, 4-D positively).

### Auxin *per se* does not quell the induction of defence genes by harpin

**Fig 2:**
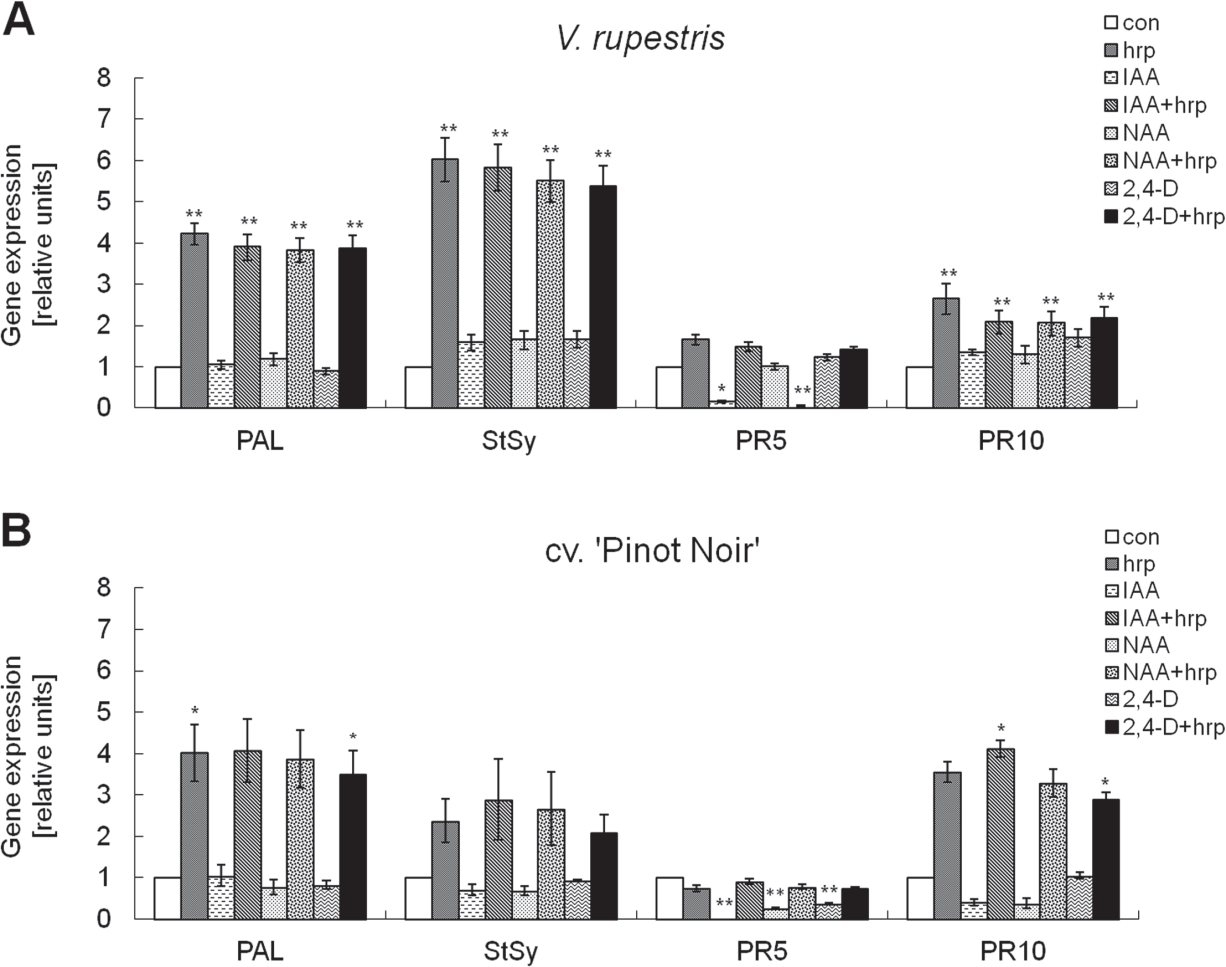
Effect of auxins on the induction of defence genes by harpin. Expression of selected defence genes was conducted by semiquantitative RT-PCR in response to harpin (hrp, 9 μg ml^-1^), auxins (IAA, NAA or 2, 4-D, 50 μM), or auxins combined with harpin as compared to ethanol as solvent control (con) in *V. rupestris* (**A**) and cv. ‘Pinot Noir’ (**B**). Transcript abundance was analysed 30 min after addition of harpin preceded by incubation for 60 min with the respective auxins. Genes included *StSy*, stilbene synthase; *PAL*, phenylalanine ammonia lyase 1; *PR*5, *PR*10, pathogenesis-related proteins 5 and 10). Quantification of transcripts were calculated relative to elongation factor 1α (EF1 α) as internal standard. The data represent mean values from three independent experimental series; error bars show standard errors. Expression difference of defence gene as compared to solvent control were analyzed using ANOVA with * significant at *P* = 5%, and ** significant at *P* = 1%.

A previous study has found for tobacco leaves that auxin can inhibit local and systemic immunity without affecting defence-related genes [30]. We therefore selected *StSy*, *PAL*, *PR*5 and *PR*10 as marker genes to investigate the effect of exogenous auxin on gene expression mediated by the bacterial elicitor harpin. As time point we selected 30 min after addition of harpin, because we knew from previous time-course studies [8] that the transcriptional response was already fully achieved at this time point. In both *V*. *rupestris* and cv. ‘Pinot Noir’, the natural auxin IAA did not significantly induce transcripts of *StSy*, *PAL* and *PR*10, and also did not affect harpin-triggered *StSy* and *PAL* expression (Fig 2). The effect of NAA and 2, 4-D was very similar to IAA without significant inhibition of *StSy*, *PAL* and *PR*10 transcripts. In contrast to IAA and NAA, application of 2,4-D induced *PR*10 expression somewhat, but this induction was not significant. Compared to control, addition of three auxins alone significantly down-regulated *PR*5 transcripts especially in cv. ‘Pinot Noir’, whereas harpin-triggered *PR*5 expression levels remained basically unaltered. These modulations of transcription show that the auxin treatments were biologically active and specific. However, none of these auxin treatments was able to significantly alter the induction of defence genes by harpin.

### Auxin inhibits harpin-induced cell death

**Fig 3:**
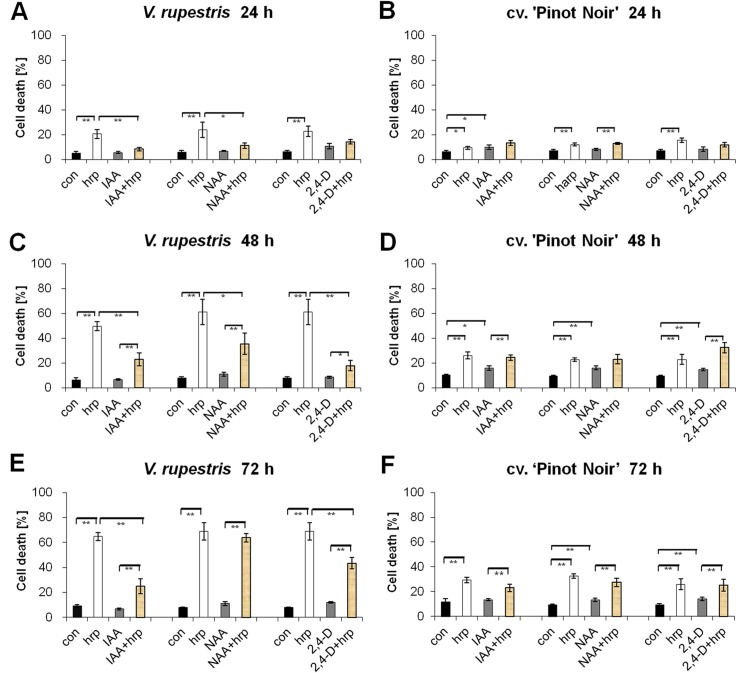
Effect of auxins on harpin-induced changes of viability. Following subculture weekly, cells were treated with 50 μM of the three auxins IAA, NAA and 2,4-D, with 9 μg ml^-1^ harpin (hrp), or with combinations of harpin with the auxins in comparison to ethanol as a solvent control (con) in *V*. rupestris (**A**, **C**, **and E**) and cv. ‘Pinot Noir’ (**B**, **D**, **and F**). Data show mean and standard errors from three independent experiments. Brackets indicate significance levels of differences using ANOVA with * significant at P = 5%, and ** significant at P = 1%.

Gopalan (2008) reported for tobacco that hypersensitive cell death initiated by harpin could be reversed till a very late stage by auxins [30]. Also, both grapevine cell lines responded to harpin treatment by induction of cell death, and this response could be quelled by pretreatment with auxins. Compared with the solvent control, IAA, NAA, and 2,4-D by themselves, induced almost no cell death in *V*. *rupestris* (Fig 3A, 3C and 3E), but triggered a small, significant elevation of cell death in cv. ‘Pinot Noir’, reaching a maximum of almost 15% at 48 h followed by a gradually decrease at 72 h (Fig 3B, 3D and 3F). However, harpin caused a strong increase in cell death in *V*. *rupestris* up to almost 60% at 72 h (Fig 3E), and this induction of cell death was significantly reduced when auxins were applied together with harpin, especially for IAA and 2,4-D (Fig 3A, 3C and 3E). In contrast, in cv. ‘Pinot Noir’, the induction of cell death by harpin, although significant, was less pronounced and therefore also the fact that in the presence of the three auxins cell death remained at a low level was less conspicuous compared to *V*. *rupestris* (Fig 3B, 3D and 3F). These findings are consistent with Gopalan (2008), where auxin was shown to suppress harpin-triggered cell death in tobacco [30], indicating that auxin also acts as a negative regulator in ETI-like cell death in *V*. *rupestris*.

### Actin is involved in auxin-dependent modulation of harpin-induced cell death

**Fig 4:**
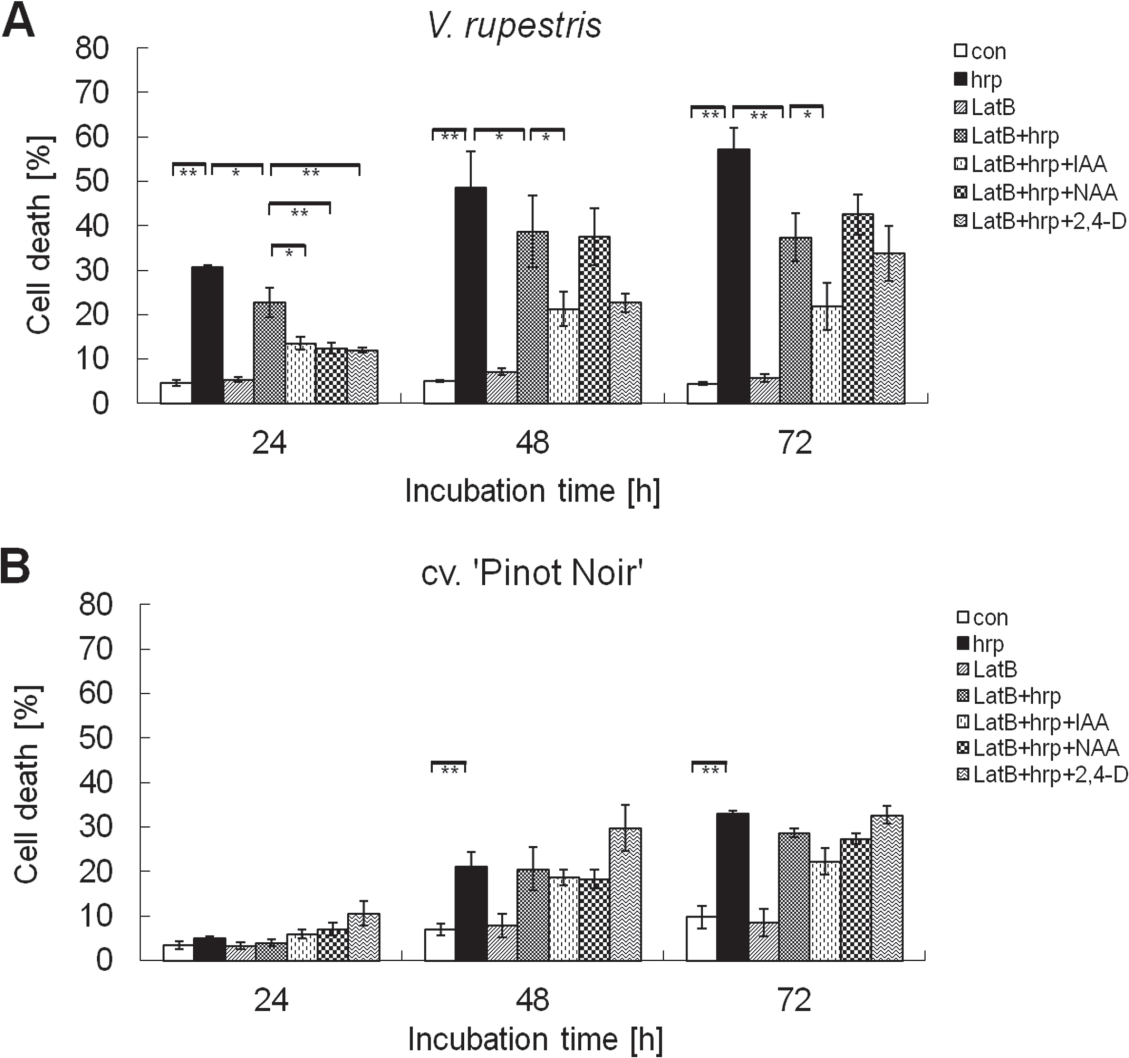
Effect of Latrunculin and auxins on harpin-induced changes of cell viability. Cells were treated with Latrunculin B (Lat B, 2 μM), with harpin (hrp, 9 μg ml^-1^), or with LatB in combination with harpin and auxin (IAA, NAA or 2, 4-D, 50 μM) following subculture weekly, versus ethanol as solvent control (con) in *V. rupestris* (A) and cv. ‘Pinot Noir’ (B). Data show mean and standard errors from three independent experiments with 500 cells. Significance levels of differences was analyzed using ANOVA with * significant at P = 5%, and ** significant at P = 1%.

It has been shown that auxin transport is dependent upon actin filaments, and application of exogenous auxin can induce debundling of actin filaments [16]. Since bundling of actin is an early and necessary event in plant cell death [14], the inhibition of harpin-induced cell death by auxin (Fig 3) might be caused by auxin-dependent remodelling of actin. To investigate this possibility, we asked, whether pharmacological manipulation of actin would interfere with harpin-triggered cell death as well. We used Latrunculin B (LatB), a drug that irreversibly sequesters G-actin and thus impinges on actin filaments depending on their innate turnover. Application of LatB alone did not trigger cell death, neither in *V*. *rupestris* (Fig 4A), nor in cv. ‘Pinot Noir’ (Fig 4B). However, LatB significantly suppressed harpin-triggered cell death from 24 h in *V*. *rupestris* (Fig 4A), but hardly in cv. ‘Pinot Noir’. Furthermore, the combined effect of LatB and three auxins on harpin-induced cell death was monitored. It was clearly seen that LatB combined with auxins suppressed harpin-triggered cell death at 24 h in *V*. *rupestris*. LatB in combination with the different auxins was not able to enhance the amelioration reached by the auxins alone, especially prominent for NAA (compare Figs 4A and 3C). In cv. ‘Pinot Noir’, where harpin-triggered cell death was less pronounced, as well as the effects of the different auxins (Fig 3B, 3D and 3E), the combination of LatB with these auxins yielded only marginal effects compared to treatment with harpin alone. These results support a scenario where actin assembly participates in harpin-induced cell death, providing a partial phenocopy of the auxin-dependent modulation of harpin-induced cell death.

### Harpin-induced actin bundling is reversed by auxin in a phospholipase D dependent manner

**Fig 5:**
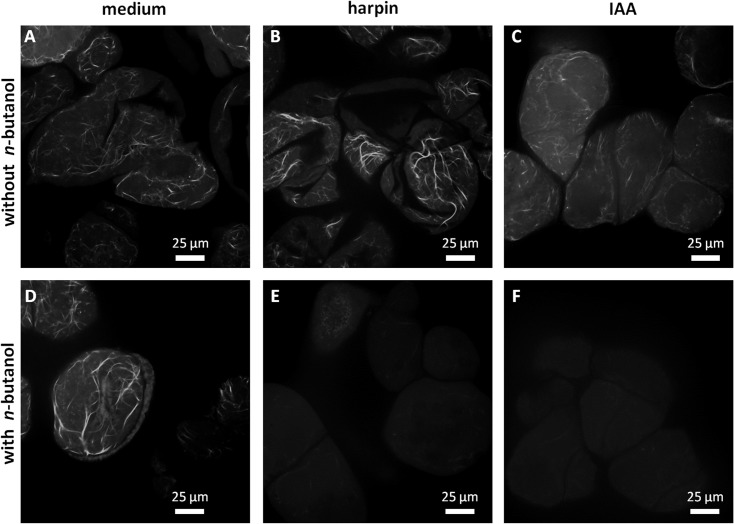
Response of cortical actin filaments to harpin, auxin, or n-butanol, an inhibitor of phospholipase D in *V*. *rupestris*. Representative confocal sections in the cortical region after visualisation of actin by fluorescent phalloidin following incubation for **A**,**D** 30 min in cultivation medium (); **B,E** 30 min in medium supplemented with 9 μg.ml^-1^ harpin; **C,F** 30 min in medium complemented with 10 μM of the natural auxin indole-3-acetic acid. **A-C** without *n*-butanol preincubation, **D-F** with n-butanol preincubation (30 min, 0.5% (v/v) *n*-butanol.

**Fig 6:**
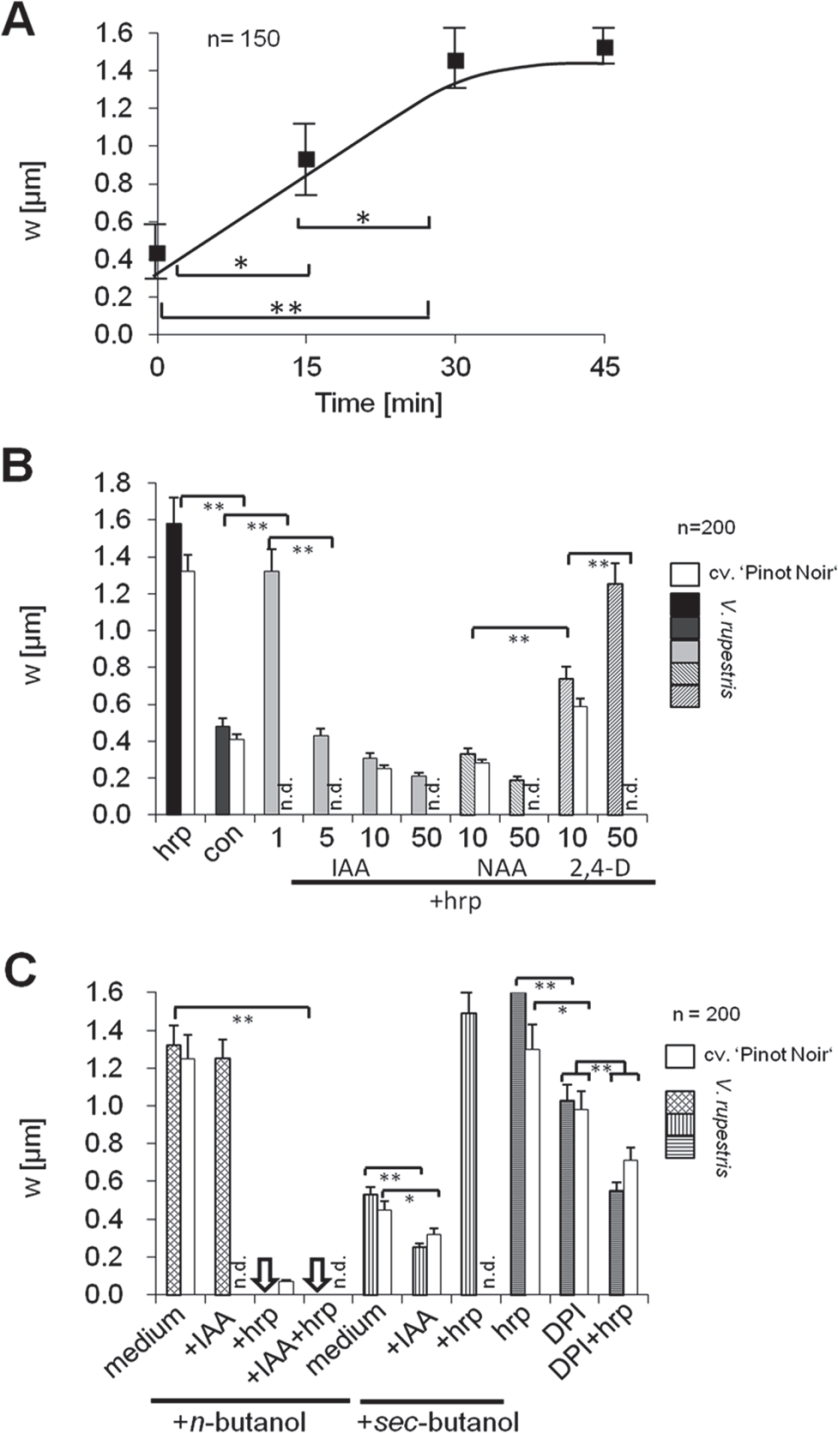
Quantification of actin responses by measuring apparent width (w) of actin bundles. **A** Time course of actin bundling in response to 9 μg^.^ml^-1^ harpin. **B** Dose-dependency of harpin-induced actin bundling over different concentrations of either the natural auxin indole-3-acetic acid (IAA), or the artificial auxins 1-napthyl acetic acid (NAA), or 2,4-dichlorophenoxy acid (2,4-D) as compared to the control (con) or harpin (hrp) in the absence of supplemental auxins measured after 60 min of incubation in *V*. *rupestris* and cv. ‘Pinot Noir’. Concentrations of auxins indicated in μM. "n.d." means "not determined" to indicate that this value was not measured in cv. ‘Pinot Noir’ (to distinguish it from a zero value). **C** Effect of pharmacological inhibition of phospholipase D by preincubation with 0.5% (v/v) of *n*-butanol or the inactive analogue *sec*-butanol for 30 min prior to the indicated treatment for 30 min, and effect of pharmacological inhibition of RboH on harpin-induced bundling by preincubation with 20 μM dephenylene iodonium (DPI) for 30 min prior to the indicated treatment for 30 min. **IAA** 10 μM indole-3-acetic acid, **hrp** 9 μg^.^ml^-1^ harpin. Arrows represent complete elimination of actin, such that no value for bundling could be measured. **n** represents the number of individual cells per sample, error bars standard errors for a population randomly collected from three biological replicates and 200 cells were used for each experiment. Brackets indicate significance levels of differences with * significant at *P* = 5%, and ** significant at *P* = 1%. "n.d." means "not determined" to indicate that this value was not measured (to distinguish it from a zero value).

To further assess the effects of harpin and auxins on the organisation of cortical actin, microfilaments were visualised by fluorescent phalloidin and single sections recorded by spinning-disc confocal microscopy in the cortical cytoplasm. To exclude differences caused by cell-cycle dependent actin remodelling, cells were probed during expansion phase (as for the other experiments). As shown for representative treatments (Fig 5), the cortical actin altered its degree of bundling depending on the treatment (a quantification of this effect is given in Fig 6). In control cells incubated for 30 min in cultivation medium, a meshwork of filaments was observed emanating from more bundled cables (Fig 5A). Following incubation for 30 min with the usual concentration of harpin (9 μg ml^-1^), the cables were clearly predominant and also had become thicker (Fig 5B), which was more pronounced in *V*. *rupestris* than that in cv. ‘Pinot Noir’ (Fig 6). In contrast, for incubation with 10 μM of IAA (in absence of harpin) such bundles were absent (Fig 5C).

To test, whether these responses were dependent on the activity of phospholipase D, we used a pretreatment with 30 min of the inhibitor *n*-butanol [0.5% (v/v)]. The pretreatment by itself caused actin bundling that was comparable to that induced by the harpin treatment (compare Fig 5D with Fig 5B). Interestingly, harpin added following this *n*-butanol pretreatment eliminated actin filaments completely, such that almost no F-actin was detectable upon phalloidin staining (Fig 5E), and this effect could not be prevented, when 10 μM IAA were added together with harpin (Fig 5F). These cells died soon afterwards.

To characterise these effects on a statistical basis, average apparent thickness of actin cables was quantified as described in Nick *et al*. (2009) [25] following variations of treatment parameters. First, the time course of harpin-induced actin bundling was followed (Fig 6A). Bundling had significantly increased already at the first assessed time point 15 min after addition of harpin, and increased further until reaching saturation at 30 min. This harpin-induced bundling was suppressed, when simultaneously the natural auxin indole-3-acetic acid (IAA) was administered (Fig 6B), whereby already 1 μM of auxin produced a slight (but still insignificant) suppression, and 5 μM of auxin could decrease bundling strongly down to the level observed in the control. The transportable artificial auxin NAA was similarly in the suppression of harpin-induced bundling. In contrast, the non-transportable artificial auxin 2,4-D given at 10 μM was significantly less efficient than the other two auxins, and at 50 μM of 2,4-D actin bundles were only insignificantly thinner as compared to a treatment by harpin alone.

As mentioned above, a pretreatment with *n*-butanol, an inhibitor of the phospholipase D caused actin bundling, and this bundling was almost as strong as that induced by harpin (Fig 6C). However, whereas the harpin-induced bundling could be efficiently suppressed by 10 μM of IAA (Fig 6B), *n*-butanol induced bundling was persistent to auxin (Fig 6C). Harpin administered following the *n*-butanol treatment eliminated actin filaments completely, such that the values for bundle thickness became zero (Fig 6C, arrows). In contrast to *n*-butanol, pretreatment with *sec*-butanol, an inactive analogue, left actin responses unaltered, both with respect to harpin induced bundling as with respect to auxin-induced debundling (Fig 6C, white bars). In a different set of experiments, we also probed for the influence of RboH by preincubation with the specific inhibitor DPI that, in previous studies had been shown to suppress harpin-induced gene activation [7]. Interestingly, preincubation by DPI alone induced some bundling of actin that was significant, although it did not reach the same level as that induced by harpin (Fig 6C, horizontally striped bars). Although both, DPI and harpin, given alone, induced actin bundling, their combination produced actin filaments that looked fairly normal and were also with respect to their degree of bundling comparable to the control situation.

### Auxin quells Harpin-induced oxidative burst

**Fig 7:**
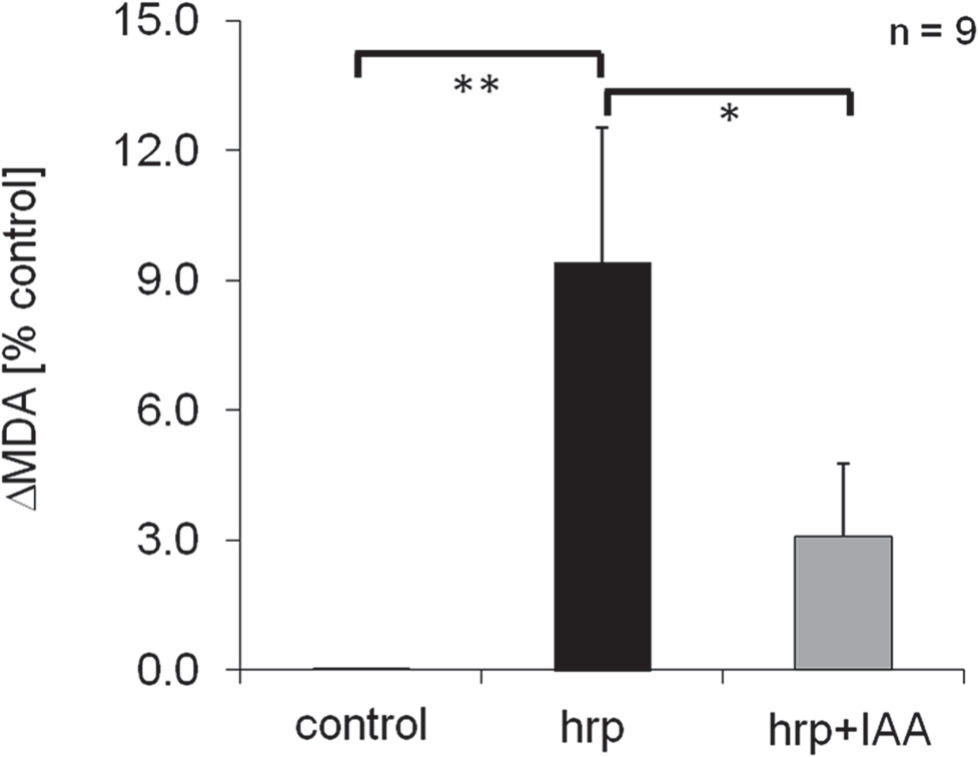
Auxin quells the stimulation of oxidative burst induced by harpin. Concentrations of the lipid peroxidation product malone dialdehyde (MDA) were determined as readout for superoxide-activity in cells of V. rupestris either treated with harpin alone or following pretreatment with 50 μM IAA for 1 h. The response was scored 30 min after addition of 9 μg ml^-1^ harpin. As negative control, cells were incubated with culture medium. Values represent means and standard errors from three independent biological replicas for the increase in MDA over the control. Brackets indicate significance levels of differences with * significant at *P* = 5%, and ** significant at *P* = 1% as tested by a paired t-test.

To test, whether auxin can modulate the oxidative burst triggered by harpin, we probed for potential changes of superoxide content using malone dialdehyde (MDA) as readout, a degradation product from lipid peroxidation triggered by partially reduced oxygen species (mainly superoxide). Compared to the control, harpin induced a significant increase of MDA (Fig 7) in *V*. *rupestris* indicative of a stimulation of oxidative burst, whereas this increase was almost eliminated by pretreatment with IAA.

## Discussion

### Auxin modulates specific branches of defence signalling

In order to remain competitive, vegetative plant development is optimised for rapid growth. However, to cope with unfavourable conditions—may it be abiotic or biotic stress—resources have to be repartitioned in a balanced and regulated manner [18]. Auxin as major regulator for growth is therefore expected to act as negative regulator of defence.

In fact, different events in auxin synthesis, signalling, transport as well as catabolic metabolism have been shown to be modulated in the context of plant defence [27,31]. As to be expected, bacterial pathogens can produce their own auxin to use it as effector in order to quell plant defence, and plants can restore defence by specific miRNAs to interfere with auxin signalling [32]. Exogenous auxin can promote disease symptoms as shown for the interaction between *Arabidopsis* and *P*. *syringae* [33], *Arabidopsis* and *B*. *cinerea* [34], rice and *X*. *oryzae* [35], tobacco and *B*. *cinerea* [36], or sweet orange and *X*. *axonopodis* [37]. In the current study, we use a cellular model in grapevine to dissect the cellular events underlying this modulation of defence signalling by auxin.

We observe that the natural auxin IAA can efficiently suppress cell death induced by the elicitor harpin. This reduction can be mimicked by the actin assembly blocker Latrunculin B. Together with the finding that harpin-induced actin bundling can be reversed by auxin (Fig 6), this supports a model, that the modulation of harpin inducible cell death by auxin is associated with actin reorganisation. In contrast, induction of defence genes and early apoplastic alkalinisation as markers for defence signalling are not quelled by auxins. Thus, harpin-triggered signalling seems to be bifurcated into a branch that is modulated by auxin, and a branch that is not. It is the auxin-sensitive branch that conveys cell-death signalling and also involves actin. The induction of the defence genes *PAL*, *StSy*, *PR*5, and *PR*10 seems to be conveyed by the auxin-insensitive branch. This leads to the question, whether these different branches are linked with the two layers of plant-innate immunity [1]

### Auxin leaves basal defence unaltered

The basal layer of innate immunity (so called PTI) [1] that can be triggered for instance by the PAMP flg22 involves a rapid influx of calcium that can be monitored by a rapid alkalinisation of the apoplast and is followed by activation of a MAPK cascade culminating in the induction of defence genes [38]. These events have been also observed in the grapevine cell models used in the current study in response to flg22 and were mapped with respect to time course and amplitude [8]. Targets of this signalling are metabolic genes involved in the synthesis of stilbenes that act as phytoalexins in grapevine. All of these events can be triggered by harpin as well. However, in addition, harpin, induces a rapid oxidative burst and programmed cell death. In the current study we asked, whether the response of basal defence (i.e. those events that are activated by both flg22 and harpin) can be modulated by auxin. However, for none of the three auxins (IAA as natural auxin, NAA and 2,4-D as artificial auxins), there was a significant modulation of the tested defence genes (*PAL*, *StSy*, *PR*5 and *PR*10).

Apoplastic alkalinisation is often used as early and convenient readout for the activity of the calcium influx activating basal defence [39] and did show certain modulations. However, we did not observe a temporal shift of rapid alkalinisation, but rather changes in the amplitude of the sustained change in pH. These changes are to be expected from auxin-induced modulation of proton pumps situated in the plasma membrane that are thought to play a role in growth-related relaxation of the cell wall [40]. For the rapid expression of defence genes, these sustained changes of pH are most likely irrelevant, because they occur later. What is relevant is the onset of alkalinisation, because this part of the time course reports on the activity of defence-related calcium influx: Interestingly, in *V*. *rupestris*, IAA advanced alkalinisation, and thus acts antagonistically to the expected acidification of the cell wall (Fig 1A). This advance was observed for 10 μM IAA (which corresponds to the optimum in the bell-shaped dose-response characteristic for natural auxins) [26], but hardly detectable for a superoptimal concentration of 50 μM IAA. The artificial auxins NAA (stable, transportable), and 2,4-D (stable, non-transportable) did not advance the response, but merely reduced its amplitude, which is probably the consequence of sustained auxin-induced activation of the plasma membrane located proton ATPases by these stable artificial auxins. The accelerated harpin-triggered alkalinisation by 10 μM IAA might be related to auxin-triggered release of actin tension below the membrane that should amplify the activity of mechanosensitive calcium channels [41]. This hypothesis is also consistent with our previous observation that Latrunculin B can amplify alkalinisation [8]. What we did not observe, was a delay in the onset of harpin-triggered alkalinisation. Alkalinisation initiated at the same time point throughout, no matter how the different auxin treatments subsequently modulated the amplitude of the response. This was consistent with the absence of any significant auxin effect on the accumulation of the tested defence-related transcripts. Thus, basal defence, as far as we have tested it, appeared to proceed independently of the auxin treatment.

### Auxin modulates cell-death related defence through dynamic actin

In addition to general basal immunity leading to the accumulation of defence metabolites, plants have evolved a second layer of immunity that culminates in programmed cell death, a strategy that is especially efficient for the containment of biotrophic pathogens [1]. This layer of defence can be elicited by treatment with harpin [8]. However, it can be efficiently produced only in cells of *V*. *rupestris* but not in cv. ‘Pinot Noir’ (Fig 3), consistent with previous results indicating that this response is clearly dependent on genotype [6,8]. A similar difference between the two cell lines is also observed with respect to the amplitude of the actin response (Fig 6), indicating that the difference between the cell lines must be located upstream in defence signalling. In fact, both lines differ in the amplitude of apoplastic alkalinisation evoked by harpin treatment [6] as very early readout of defence indicating that either number or activity of the calcium-influx channels that underlies this pH response, is reduced in cv. ‘Pinot Noir. Auxin modulates both, the response of actin as well as the cell-death response (Figs 3 and 6) indicating that it acts downstream of the calcium channels, but upstream of actin. The different auxins differed in their ability to quell harpin-induced cell death. The natural auxin IAA produced the most pronounced and stable effect, whereas the artificial NAA was least effective, and the artificial 2,4-D showed intermediate activity on cell death (Fig 3). The small residual concentration of 2,4-D which was present in all experiments to sustain viability of the culture cannot account for any of the observed differences, because it was present throughout, also in the controls. It seems to be necessary to activate the synthesis of endogenous IAA required to organise cellular development [6,42]. Comparative pharmacology on the signalling triggered by these two artificial auxins in tobacco study demonstrated that 2,4-D signalling involves the activation of G-proteins. In contrast, NAA signalling was independent of G-proteins [43]. The two auxins also differ with respect to their effect on actin filaments: for roots of *Arabidopsis* 2,4-D has been reported to eliminate actin (similar to Latrunculin B), whereas NAA does not [44]. On the other hand, NAA is effective in detaching transvacuolar actin cables into finer strands, an activity that is not observed for 2,4-D [45], and the same was observed for cortical actin in our experiments (Fig 6B). In this study, the auxin effect on cell death could be mimicked by a mild treatment with Latrunculin B (Fig 4), a compound that irreversibly sequesters G-actin and thus eliminates actin filaments depending on their innate turnover [46]. Latrunculin B also acted synergistically with IAA indicating that they share the same target. Thus, different auxin species control different aspects of actin organisation. These findings point to a scenario, where changes in dynamic actin filaments mediate the effect of harpin on programmed cell death.

### The actin-auxin circuit as switch between life and death?

Auxin regulates actin organisation [45], which can be perfomed by controlling actin dynamics via actin-depolymerisation factor 2 [47]. Actin, in turn, regulates auxin transport [25], constituting a self-referring oscillating circuit that underlies the regulation of cell expansion and cell division by auxin [16]. Cell-death triggering elicitors such as harpin [6], HrpZ [9], or resveratrol [7], cause a breakdown of the dynamic meshwork of cortical actin filaments that is followed by a contraction of actin cables. This actin is known to stabilize membrane integrity in a great number of systems [48], and by TIRF microscopy fluorescently labelled actin can be detected to be directly linked with the plasma membrane of plant cells [41]. A specific Networked (NET) superfamily of actin-binding proteins specify membrane compartments differentially interacting with actin [49]. This membrane-associated population of actin regulates membrane integrity [50] and dynamics [51]. Perturbations of membrane integrity followed by rapid detachment of actin and formation of actin cables participate in PTI and are regulated by actin depolymerising factor 4 in *Arabidopsis* [10,11].

**Fig 8:**
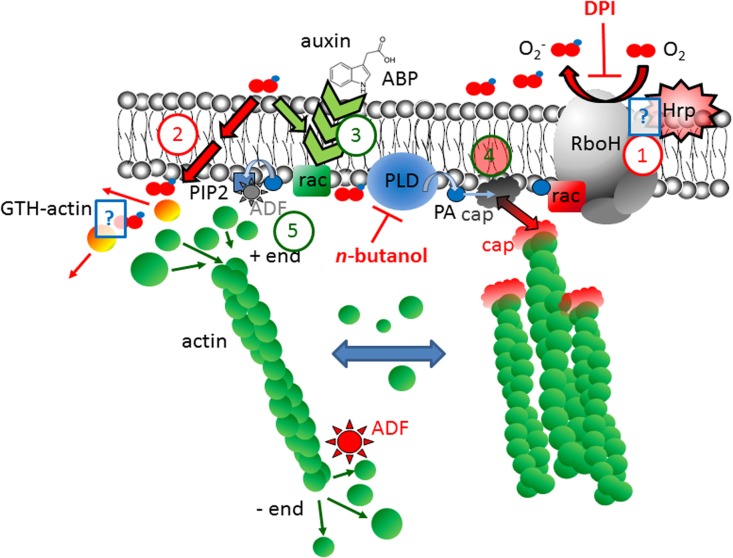
Working model on the antagonistic interaction of signalling triggered by harpin and auxin. To reduce complexity, only the earliest events are depicted, omitting ROS activation of calcium influx and effects of rac1-signalling on auxin transport. ① harpin activates the NADPH-dependent oxidase RboH leading to the production of superoxide that can spread in the apoplast. ② Superoxide can penetrate through the plasma membrane (probably by aquaporins) and glutathionylate actin in residue Cys374. This will sequester G-actin from being integrated into the growing end of actin filaments. ③ Alternatively, superoxide can be recruited to transduce the effect of auxin (perceived via the auxin-binding protein, ABP) upon the activation of phospholipase D (PLD) through the small G-protein Rac. ④ PLD will generate phosphatidic acids (PA) that can sequester actin capping proteins (cap) to the membrane, such that elongation of actin filaments is enabled. Alternatively, PA can be partitioned to recruit Rac for the activation of the RboH complex. In this case, the capping proteins will not be recruited to the membrane and constrain the elongation of actin filaments leading as secondary consequence to the formation of thick cables through the activity of severing proteins in combination with free G-actin the formation. ⑤ As third alternative, PA can be converted to PIP2, which will recruit actin-depolymerization factor (ADF) to the membrane. Since ADF is sustaining the monomer turnover at the minus end of actin filaments, this recruitment results in a higher stability of fine cortical actin filaments. The molecular targets for the inhibitors diphenyliodonium (DPI), and *n*-butanol are inserted in red. Hypothetical aspects of the model that have not been addressed experimentally in plant cells, are indicated by blue question marks: The interaction of Harpin with RboH (①) has not been addressed experimentally so far. Also the glutathionylation of actin in consequence of superoxide penetration (②), so far has been shown for animal systems, but not been addressed in plant cells.

The observed antagonism between auxin and harpin-induced defence signalling, and the response of actin in response to pharmacological manipulation of RboH and PLD was integrated with the published records into a working model of molecular interactions at the plasma membrane being aware that such a model must be a strongly simplified projection of the complex and dynamic reality: harpin activates an oxidative burst [8], and requires the NADPH-dependent oxidase RboH to activate defence genes such as stilbene synthase [7]. Although the molecular identity of the receptor for harpin remains to be elucidated, it is possible to estimate the abundance of the putative binding site by kinetic analysis to be in the μM range [6], which indicates that the binding site must be a relatively abundant. Binding of harpin to this receptor activates RboH (Fig 8, ①) resulting in accumulation of superoxide anions in the apoplast. However, so far, the interaction of harpin with RboH has not been addressed experimentally. The diffusion of superoxide anions can activate calcium influx, which can be monitored by apoplastic alkalinisation [6,8]. This calcium peak will activate actin severing proteins such as gelsolins [51]. However, superoxide anions can also permeate the plasma membrane and interact with targets in the cortical cytoplasm.

One of these targets is actin itself: Oxidative stress will cause glutathionylation of a critical cysteine residue in position 374 of actin and the glutathionylated actin (Fig 8, ②) will be sequestered from polymerisation [53,54]. Also in plants, oxidative stress results in glutathionylation of actin [55], suggesting that this mechanism has been conserved through eukaryotic evolution. However, it has still to be demonstrated, whether this modification of actin is caused by superoxide anions generated by RboH as depicted in the model. The sequestration of G-actin will progressively eliminate dynamic filaments due to their innate turnover, whereas non-dynamic filaments or cables will persist and therefore become dominant.

However, actin is not the only target for cytoplasmic superoxide—this oxidative species (in concert with or upon transformation into the messenger NO) is also recruited to transduce the activation of phospholipase D (PLD) through small GTPases of the Rac family [56]. This event is integrated into auxin signalling (Fig 8, ③). In other words, there is a mutual competition of both signalling pathways for superoxide as shared signal. By the way, also the use of this small oxidative species as transducer of Rac GTPases seems to be an ancient motif of signalling, since it has been originally discovered in the context of cell migration in mammalian cells [57,58].

The activation of phospholipase D will produce phosphatidic acids (PA) as downstream messengers. Again, there is quite some branching with respect to downstream targets (for review in animal systems [59]; for review in plant systems [60]): (i) PA can sequester actin capping proteins, which will release elongation of the highly dynamic cortical filaments subtending the plasma membrane [61]. (ii) PA can also be recruited to partition Rac1 for activation of RboH [62]. (iii) PA can be further converted to PIP2, which will sequester actin-depolymerization factors (ADF) to the membranes [17], which will strongly reduce treadmilling at the minus ends of actin treadmilling by a factor of 25 [63]. Since both, process (ii) and process (iii) compete for PA, cortical actin filaments will be either in a state, where they elongate, but at the same time treadmill at their minus end, or a state, where they stop elongation, but remain stable due to suppressed treadmilling at their minus end. In addition, there will be a balance between auxin dependent actin-related targets of PA and recruitment of Rac1 and PA for the activation of RboH.

Although this (simplified) working model might appear complex at first sight, the central points are straightforward: by mutual competition of the auxin and the harpin triggered pathways for superoxide (first level), for Rac (second level), and for PA (third level), the two pathways will act antagonistically. In the following, the observed actin responses will be explained on the base of this model:

Under control conditions, RboH will provide a ground activity that will yield a low, but not negligible abundance of superoxide that will be available for signalling triggered by endogenous auxin (Fig 8, ③). There is some PLD activity producing a ground level of PA and a smaller amount of PIP2. As a consequence, there will be an equilibrium of capped and uncapped cortical actin filaments with a relatively dynamic turnover.

In response to harpin, an excess of superoxide will be formed and produce glutathionylated actin (Fig 8, ①). This will cause dynamic actin filaments disappear swiftly (due to their innate turnover). The activation of RboH will also cause a depletion of Rac and PA, which are repartitioned towards oxidative burst (Fig 8, ④), such that capping proteins (Fig 8, ④) and ADF (Fig 8, ⑤) will detach from the membrane and eliminate cortical actin, because elongation is suppressed by capping, and monomer loss is accelerated by free ADF at the minus end. The ROS-triggered calcium influx [6,8] will activate actin severing proteins [52], and the simultaneous excess of G-actin from the decay of cortical filaments will therefore produce bundling of actin cables [57].

In response to auxin, the activation of Rac will, by use of the low ground levels of superoxide from the basal activity of RboH, activate PLD, and the formed product PA will recruit actin capping proteins, such that elongation of cortical actin filaments is stimulated (Fig 8, ④). In addition, some of the PA will be available for the conversion into PIP2, which will sequester ADF, such that the cortical actin filaments will be stabilised and predominate the formation of bundled cables of actin (Fig 8, ⑤). Since the activity of RboH is low, Rac remains mainly recruited for PLD. When now, in addition, RboH is activated by simultaneous application of Harpin, a part of the Rac will be repartitioned and this should result in a balance between dynamic filaments and actin cable, which is more or less the basal situation under control conditions.

When PLD is blocked by *n*-butanol, this will cause a depletion of PA and PIP2, such that the actin capping proteins and ADF are released from the membrane, mimicking the effect of harpin with respect to actin bundling. If now, on the base of *n*-butanol pretreatment, harpin is added, this will impact actin even more drastically up by the formation of glutathionylated actin and this combinatorial effect might be the reason, why after combined treatment with harpin and *n*-butanol no actin filaments could be detected. Since auxin is acting on actin through PLD-dependent generation of PA and PIP2, a pretreatment with *n*-butanol cannot rescue the harpin triggered elimination of actin (in contrast to the situation without *n*-butanol pretreatment).

A non-intuitive prediction by this model is that inhibition of the ground activity of RboH using the specific inhibitor DPI will produce some actin bundling as well. This can be explained, because the activation of PLD by Rac requires some superoxide and will be impaired after DPI treatment. Instead, a combinatorial treatment of DPI with harpin will generate a reduced level of superoxide, but apparently sufficient amounts to convey the signal from Rac to PLD, thus establishing a more or less normal dynamics of actin, comparable to the situation in the control.

A second prediction by this model is that auxin should quell RboH-dependent oxidative burst by titration of superoxide anions for its own signalling. We have tested this prediction by measuring malone dialdehyde (MDA) levels as readout for superoxide mediated lipid peroxidation (Fig 8). As predicted by the model, we observe that the stimulation of MDA formation is quelled by pretreatment with IAA (Fig 7).

In fact, the link between actin remodelling and programmed cell death is also well supported across eukaryotic cells in general [12,13], and for plant cells in particular [14,15]. The actin-auxin oscillator thus emerges as a signalling hub that can either function in the context of growth and development, or in the context of defence-related cell death. The constrained harpin response in cells treated with Latrunculin B indicates that a dynamic population of actin that represents a target for both harpin (as activator of cell death) and auxin (as inhibitor of cell death). The efficiency of different auxin species is different and correlates with their effect on actin dynamics not with their effect on actin bundling. We therefore think that it is this dynamic subpopulation of membrane-associated actin that is acting as switch between life and death. Future work will be dedicated to uncover the molecular modifications of plant actin induced by stress-related oxidative burst, such as the putative glutathionylation of actin.

What confers functional specificity to this array of actin filaments? Actin is a very conservative molecule, and the molecular differences between different actin isotypes are minor. Specificity must come from differential decoration with actin-associated proteins. In fact, using a tetrameric photoswitchable fluorescent probe (psRFP) coupled to the actin binding Lifeact domain, it is possible to detect differences in decoration between cortical and perinuclear arrays of actin [48]. Future work will therefore be dedicated to identify those actin-decorating proteins and to probe for defence-related modulations of their activity.

## Supporting Information

S1 Table List of oligonucleotide primers used for expression analysis by semi-quantatitive RT-PCR. (PDF)

S1 Fig Dose response of harpin-induced apoplastic alkalinisation to auxins. (TIF)
