# Supplementary material for: Actin as Deathly Switch? How Auxin Can Suppress Cell-Death Related Defence
Source: PLoS One. 2015 May 1;10(5):e0125498. doi: 10.1371/journal.pone.0125498 (PMC4416736; doi:10.1371/journal.pone.0125498)
Supplement: S1 Table — (PDF) [file pone.0125498.s001.pdf]

Actin as deathly switch?

How auxin can suppress cell-death related defence

Xiaoli Chang[1,*], Michael Riemann[2], Qiong Liu[2], Peter Nick[2]

S1 Table List of oligonucleotide primers used for expression analysis by RT-PCR.

Name	GenBank accession no.	Primer sequence 5'-3'	Reference
**EF1α**	EC959059	Sense:5'-GAACTGGGTGCTTGATAGGC-3'<br>Antisense: 5'-AACCAAAATATCCGGAGTAAAAGA-3'	Reid *et al*. (2006)
**StSy**	X76892	Sense:5'-GAAACGCTCAACGTGCCAAGG-3'<br>Antisense: 5'-GTAACCATAGGAATGCTATGTAGC-3'	Kortekamp (2006)
**PAL**	X75967	Sense:5'-TGCTGACTGGTGAAAAGGTG-3'<br>Antisense: 5'-CGTTCCAAGCACTGAGACAA-3'	Belhadj *et al*. (2008)
**PR5**	Y10992	Sense:5'-CAGCTATGCAGCCACCTTC-3'<br>Antisense: 5'-TCGAAGTTGCAGTTGGTACG-3'	Kortekamp (2006)
**PR10**	AJ291705	Sense:5'-CTTACGAGAGTGAGGTCACTTC-3'<br>Antisense: 5'-GCAATAGAACATCACAAATACTCC-3'	Kortekamp (2006)

Notes: EF1α, elongation factor 1α; StSy, stilbene synthase; PAL, phenylalanine ammonia lyase 1; PR5, PR10, pathogenesis-related proteins 5 and 10, respectively.
