# Supplementary figures and images for: Actin as Deathly Switch? How Auxin Can Suppress Cell-Death Related Defence
Source: PLoS One. 2015 May 1;10(5):e0125498. doi: 10.1371/journal.pone.0125498 (PMC4416736; doi:10.1371/journal.pone.0125498)

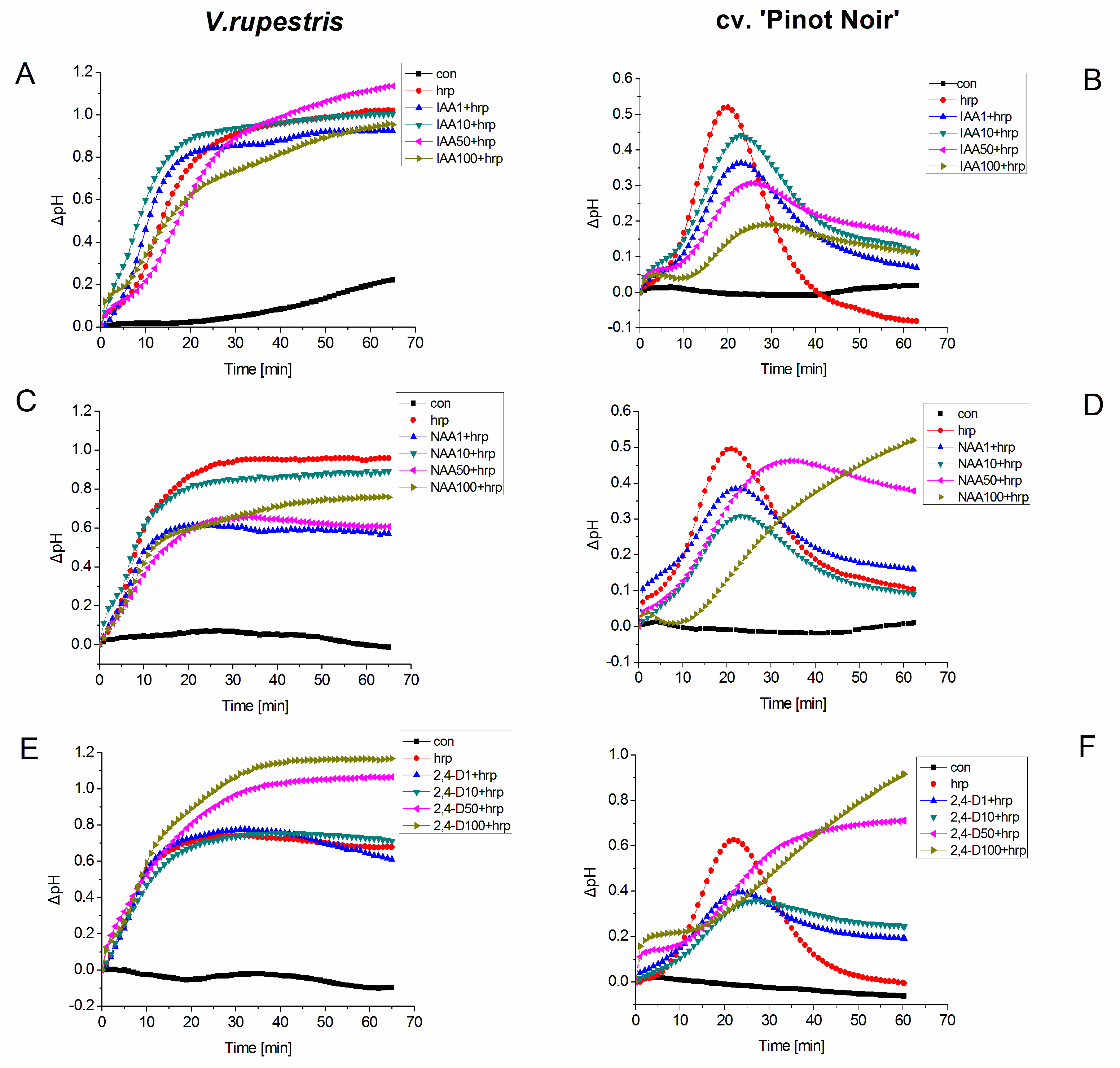

Supplement: S1 Fig — (TIF) [file pone.0125498.s002.tif]
